# Primary malignant melanoma of the cervix: A comprehensive analysis of case reports in the Chinese population

**DOI:** 10.1002/cam4.6054

**Published:** 2023-05-10

**Authors:** Yuan Ye, Aizhen Fu, Jiayi Cai, Zhen Ma, Yumei Chen, Xinxin Zou, Huiyi Li, Yongfeng Chen, Shibo Zhao, Caiyun Chen

**Affiliations:** ^1^ Department of Obstetrics and Gynecology Affiliated Hospital of Guangdong Medical University Zhanjiang China; ^2^ Department of Oncology, Zhujiang Hospital Southern Medical University Guangzhou China

**Keywords:** analysis, case reports, cervix, malignant melanoma

## Abstract

**Purpose:**

Malignant melanoma is a tumor generated from the basal melanocytes of human epidermis. Primary malignant melanoma of the cervix (PMMC) is derived from cervical melanocytes. It is an uncommon disease, mostly occurring in perimenopausal women. PMMC has a bad prognosis and lacks a defined protocol or treatment standards. The aim of this study was to analyze the impact of different surgical procedures and different adjuvant treatment modalities on their prognosis and to find risk factors for their prognosis by integrating published case report data based on the Chinese population.

**Methods and Results:**

This study included 165 patients with PMMC in the Chinese population. We used the Kaplan‐Meier method to build the survival curve, and the log‐rank test to examine the variations among the subgroups. Prognostic factors were examined utilizing the Cox proportional hazards regression model. We found that patients who underwent radical hysterectomy‐based surgery, those who underwent lymphadenectomy, and those who underwent other treatments in addition to surgery had significantly better survival rates. The overall analysis, showed that age, and FIGO Stage II, III, and IV, increased the risk of death. Moreover, radical hysterectomy (RH), total hysterectomy (TAH), lymphadenectomy, and adjuvant therapy were correlated with a decreased mortality risk.

**Conclusion:**

After summarizing the current data, we recommend radical hysterectomy, and lymphadenectomy treatment for patients with PMMC. For patients who had already undergone surgery, other treatment options had a positive effect on prognosis. For patients who had already undergone surgery, other treatment options had a positive effect on prognosis; therefore patient‐specific treatment options need to be further discussed.

## 1 INTRODUCTION

Malignant melanoma (MM) is a form of melanocytic tumor with an abnormal growth pattern. Melanocytes are formed from the neural crest, and are scattered throughout all skin and most mucosal areas. They are located in the epidermal basal layer and create the epidermal melanocyte unit. Most MM occurs on the surface of the skin, and less than 2% occurs in the female genitalia.^1^ The majority of female reproductive tract MM cases are detected in the vulva or vagina.^2^ Primary malignant melanoma of the cervix (PMMC) is extremely rare, and its prognosis is very poor.^3^ Historically, the existence of PMMC was controversial, because it was thought that the cervix was totally lacking in melanocytes. It was not until 1959 that Cid found basal melanocytes in 3.5% of cervical biopsies; this was the first demonstration of the existence of melanocytes in the cervical mucosa.^4^ Currently, there are no clear guidelines for the treatment of PMMC. We checked the guidelines for cervical cancer and none of them specifically mentioned the diagnosis and treatment of PMMC.^5^, ^6^, ^7^ Therefore, at present, clinicians still manage PMMC patients with reference to the surgical criteria for squamous cell carcinoma of the cervix, and postoperative or preoperative adjuvant treatment is empirically based. This study set out to analyze the clinical and prognostic data of patients and determine the influence of various variables on the prognosis of those patients with PMMC in the Chinese population in the past. Our results offer evidence that could be useful in the future treatment of the disease.

## 2 METHODS

### 2.1 Data sources

By searching CNKI, VIP, CSPD, and PubMed, we collected case reports/case series of PMMC in the Chinese population, from their inception until closure for this study on July 31, 2022.Keywords for the literature search were as follows: (primary malignant melanoma) AND (uterine cervix) OR (cervix) OR (cervix, uterine). Exclusion criteria included other sources of primary genital tract melanoma, genital tract melanoma of unknown source, reviews, books, and unrelated articles. In the case of duplication of study data, the studies with larger sample sizes were selected.

### 2.2 Data collection

The following information was collected: publishing year, patients age at diagnosis, patients' symptoms, Federation Internationale of Gynecologie and Obstetrigue (FIGO) stage, whether surgery was performed, surgery pattern, with or without lymph node metastases, whether more treatments were used (radiotherapy, chemotherapy, or immunotherapy), overall survival (OS) in months, and the condition of human papillomavirus (HPV) infection. Missing and unrecognizable data were marked with NA if part of the patient's clinical data was omitted from mention in the article.

### 2.3 Statistical processing

OS was based on case‐reported data, while missing and unidentifiable data were excluded from statistics. Categorical information is reported in terms of percentage and frequency. Continuous data with normal distribution are presented as mean and standard deviation (SD), while data without normal distribution are presented as medians. We used statistical software SPSS 25.0 to process the data, the Kaplan–Meier method to build the survival curve, and the log‐rank test to examine the variations among the subgroups. Prognostic factors were examined utilizing the Cox proportional hazards regression model, and *p* < 0.05 indicates statistical significance.

## 3 RESULTS

### 3.1 Search results

Following the search, 71 articles in four databases were eligible, including 165 cases. Table S1 lists the main attributes of participating patients^8^, ^9^, ^10^, ^11^, ^12^, ^13^, ^14^, ^15^, ^16^, ^17^, ^18^, ^19^, ^20^, ^21^, ^22^, ^23^, ^24^, ^25^, ^26^, ^27^, ^28^, ^29^, ^30^, ^31^, ^32^, ^33^, ^34^, ^35^, ^36^, ^37^, ^38^, ^39^, ^40^, ^41^, ^42^, ^43^, ^44^, ^45^, ^46^, ^47^, ^48^, ^49^, ^50^, ^51^, ^52^, ^53^, ^54^, ^55^, ^56^, ^57^, ^58^, ^59^, ^60^, ^61^, ^62^, ^63^, ^64^, ^65^, ^66^, ^67^, ^68^, ^69^, ^70^, ^71^, ^72^, ^73^, ^74^, ^75^, ^76^, ^77^, ^78^; these include Authors, Age, Symptom, Figo, Lymph node metastasis, Chemotherapy, Radiotherapy, Immunotherapy, Survival(m), Status, HPV infection. The main clinical manifestations were vaginal bleeding and vaginal discharge. Most cervical malignant melanoma is found in the early stage, but regardless of stage, most patients were treated by surgery. If combined with adjuvant therapy, chemotherapy was the main treatment, followed by radiotherapy, with fewer treatments involving immunotherapy. The above factors were related to patient survival.

### 3.2 Clinical data

Table 1 shows the characteristics of relevant clinical data on the 165 patients with PMMC. Of them, 16 cases were eliminated because of missing data, while the remaining were diagnosed at a median of 56 years. Among the 165 patients, 141 patients (141/165) had bleeding or discharge from the vagina; 5 patients had contact bleeding, with no symptoms, cervical nodules, cervical vegetations, hematuria, and urinary incontinence in one case each, and 14 patients had unknown clinical manifestations. According to the FIGO (2018) staging guidelines, 34 patients were Stage I, 47 were Stage II, 15 were Stage III, 7 were Stage IV, and 62 had no stage information. In all cases, 10 patients had no information on surgery, 37 did not have surgery, and the remaining 69.1% (114/165) of patients were surgically treated. Of surgeries, 53.9% (89/165) of patients received radical hysterectomy (RH), 15.2% (25/165) of patients had a total hysterectomy (TAH), and 4 patients underwent undefined surgery. In addition, of all surgical cases, 88 patients underwent lymphadenectomy while 63 patients did not undergo lymphadenectomy; corresponding information was lacking for the remaining cases. Furthermore, in all patients, 18 cases showed lymph node metastasis, while 36 cases did not; and the remaining patients had no corresponding information. Of the 18 patients with lymph node metastases, 13 underwent lymphadenectomy. Finally, 94 patients received other treatments (including radiation, chemotherapy, and immunotherapy), while 41 patients received only surgery.

**TABLE 1:** Clincal characteristics date of PMMC patients.

Clinical data		Cases	Clinical data		Cases
Symptom	VB or VD	141	Surgery	RH	89
	Contact bleeding	5		TAH	25
	Asymptomatic	1		No	37
	Cervical nodes	1	Lymph node metastasis	yes	18
	Cervical vegetations	1		no	36
	Hematuria	1	Lymphadenectomy	yes	88
	Urinary incontinence	1		no	63
Figo	I	34	III		15
	II	47	IV		7
Other treatments	Chemotherapy	87	C + I		15
	Radiotherapy	42	C + R		23
	Immunotherapy	22	R + I		1
	No	41	C + R + I		4

Abbreviations: C, chemotherapy; I, immunotherapy; R, radiotherapy; RH, radical hysterectomy; TAH, total hysterectomy; VB, vaginal bleeding; VD, vaginal discharge.

Chemotherapy was given to 87 out of the 94 patients, including 6 patients who had preoperative chemotherapy, 7 patients who had both pre and postoperative chemotherapy, 16 patients who had chemotherapy without surgery, and the remaining 58 patients received postoperative chemotherapy. Of the 42 patients receiving radiotherapy, 5 patients had preoperative radiation therapy, and 1 patient received preoperative and post‐surgical radiotherapy; the remaining 36 patients had postoperative radiotherapy. For the 22 patients who received immunotherapy, their treatments included interferon (INF), interleukin‐2 (IL‐2), Bacille Calmette‐Guerin (BCG), and anti‐PD‐1.In total, 43 patients received more than one treatment in addition to surgery. Notably, among these 43, 15 cases were treated with chemotherapy + immunotherapy, 23 cases with chemotherapy + radiotherapy, 1 case with radiotherapy + immunotherapy, and 4 cases with chemotherapy + radiotherapy plus immunotherapy.

### 3.3 Patient prognosis and survival rates

**FIGURE 1:**
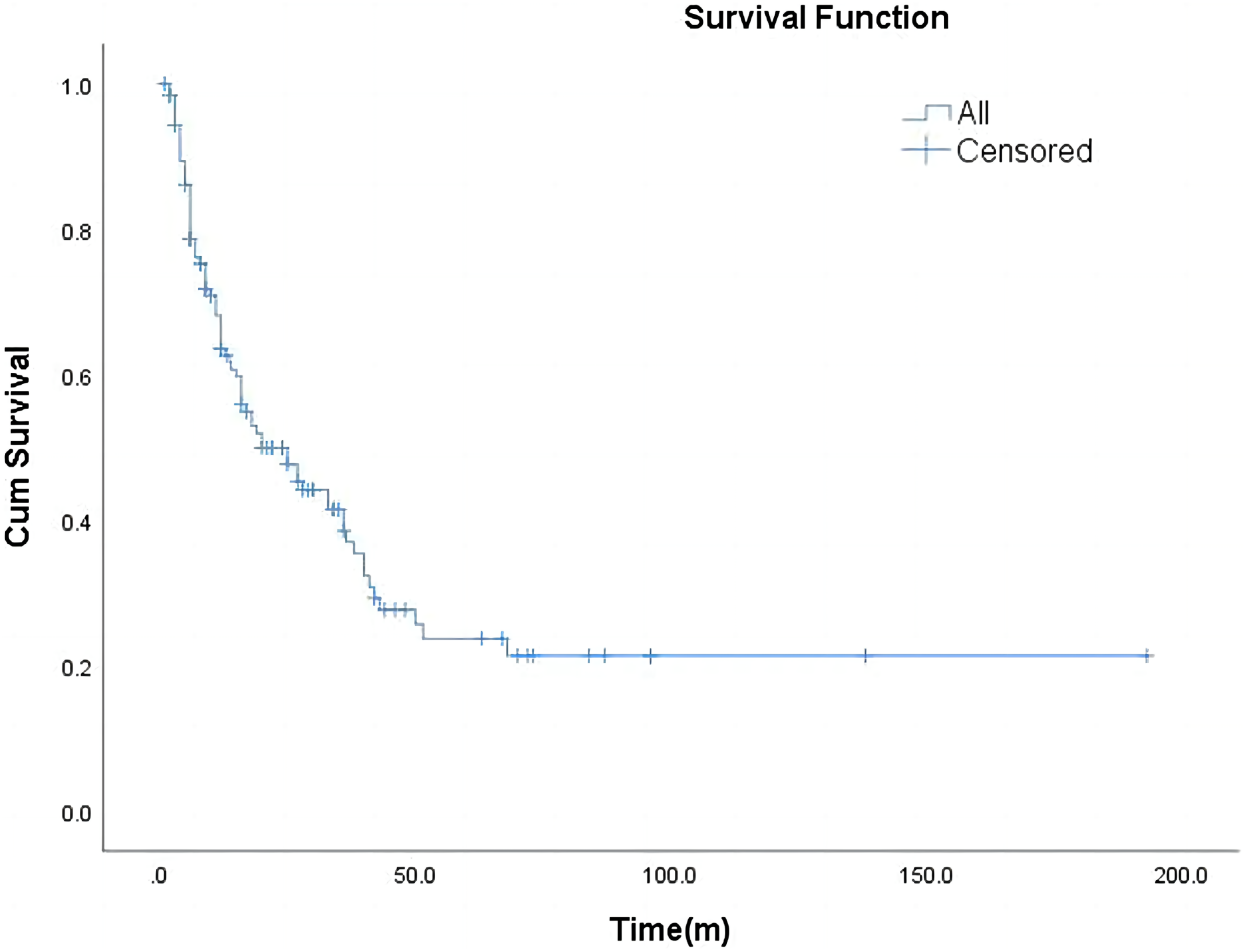
Kaplan–Meier survival curve for OS of patients. The median OS was 20 months.

**FIGURE 2:**
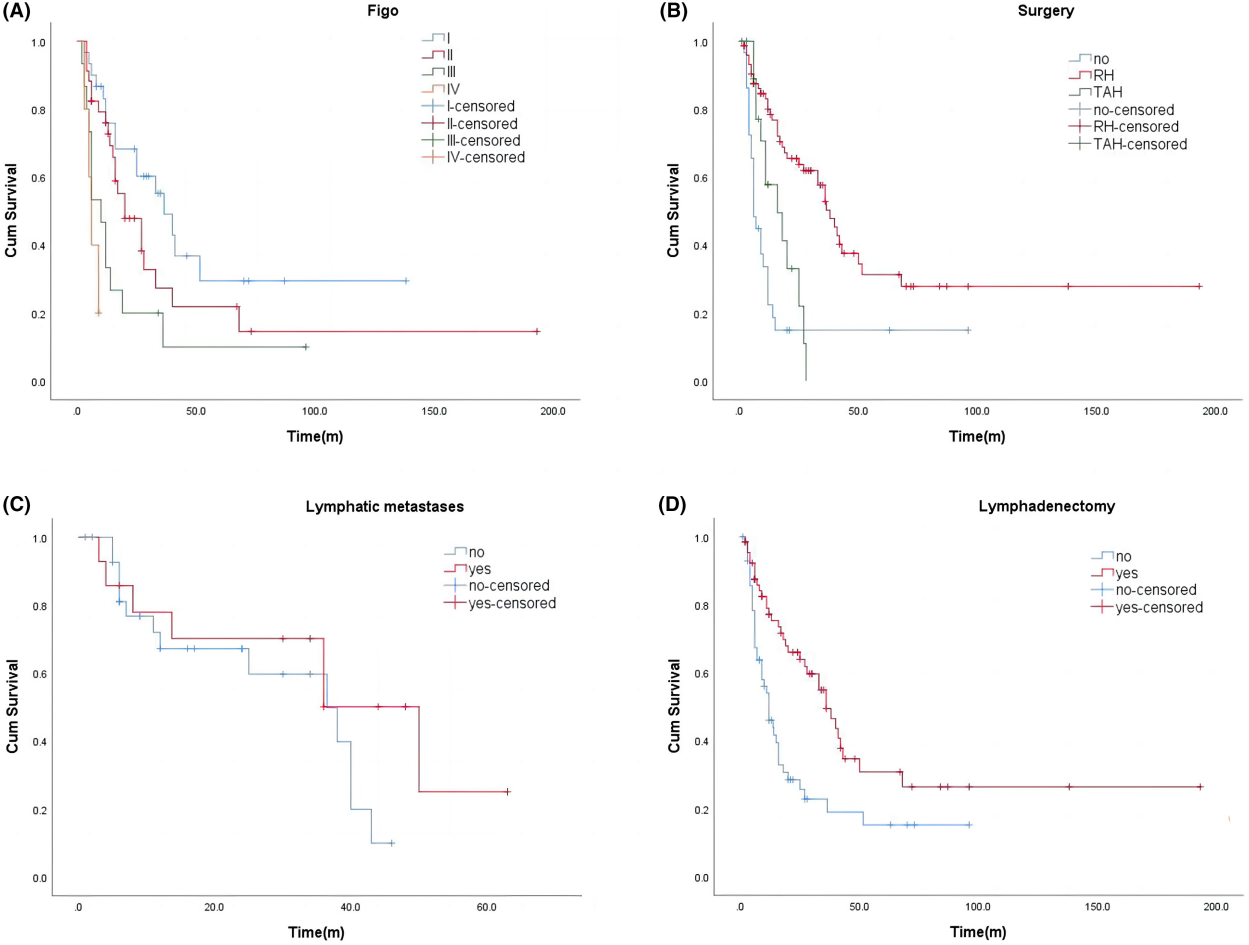
(A) Kaplan–Meier survival curves for OS in patients of different FIGO stage. The median OS of Figo Stage I, II, III and IV were 36 months, 20 months, 10 months and 6 months, respectively. (B) Kaplan–Meier survival curves for OS in patients of no surgery, TAH‐based surgery, and RH‐based surgery. There was a significant difference (*p* < 0.001) in the median OS between patients without surgery and those who received RH‐ and TAH‐based surgery, which were 6 months, 38 months, and 16 months, respectively. (C) Kaplan–Meier survival curves for OS in patients of no lymphatic metastases and Lymphatic metastases. The median OS of patients with lymph node metastasis and those without lymph node metastasis was 50 months and 36.5 months, respectively (*p* = 0.272). (D) Kaplan–Meier survival curves for OS in patients of no Lymphadenectomy and Lymphadenectomy. Patients who received lymphadenectomy had better outcomes than those who did not, with a significant difference (*p* = 0.001), with median OS of 36 months and 12 months, respectively.

Survival time was available for 126 patients, and status information for 50 patients that had survived; 76 patients died (1–193 months). There was no corresponding information for 39 patients so their survival status could not be determined. OS was assessed by generating Kaplan–Meier survival curves, and the median OS was 20 months (Figure 1). We then used the log‐rank test with stratified covariates (FIGO stage, whether surgery was performed, extent of surgery, lymph node metastasis, lymphadenectomy, and adjuvant therapy) to explore potential prognostic factors. The results demonstrated that with the progression of staging, the prognosis of patients decreased significantly (*p* < 0.001; Figure 2A), and the mean survival times from Stage I to Stage IV were 36.5, 20, 10, and 6 months, respectively.

**FIGURE 3:**
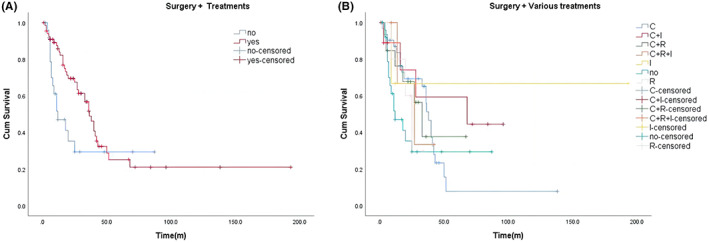
(A) Kaplan–Meier survival curves for OS in patients of surgery (RH + TAH) with and without other treatments. The median OS was 36.5 months and 12 months, respectively, and the median survival time was longer in patients receiving adjuvant therapy. (B) Kaplan–Meier survival curves for OS in patients of surgery (RH + TAH) plus various treatments vs. no other treatments. The median OS of patients who received different adjuvant treatments was not significantly different (*p* = 0.548); the median OS of chemotherapy plus immunotherapy was the longest, which was 68 months; The median OS of chemoradiotherapy, chemotherapy alone and radiotherapy alone were 33 months, 38 months and 25 months. MedianOS without any adjuvant therapy was 12 months.

To remove confounding effects of uneven diffusion of clinical features among patients at different stages, we tested whether age, lymph node metastasis, lymphadenectomy, and adjuvant treatment were the same among patients (Table S2). The findings demonstrated non‐significant variations in age, lymph node metastasis, lymphadenectomy, and adjuvant treatment among the groups (Stage I to Stage IV). Patients who did not receive surgery (median OS 6 months) had significantly poorer outcomes than those who received RH‐based and TAH‐based surgery (*p* < 0.001), while patients who underwent RH‐based surgery (median OS 38 months) had a better prognosis than for TAH‐based surgery (median OS 16 months) (Figure 2B).After the examination for differences in stage, age, and lymph node metastasis factors, the results showed a non‐significant difference in lymph node metastasis and age among the groups (RH, TAH, and no surgery), but, there was a significant difference in stage (Table S3).Although the average survival time of patients with non‐lymph node metastasis and lymph node metastasis was not statistically significant, patients with lymph node metastases had a better prognosis (*p* = 0.272; Figure 2C). However, the prognosis was better for patients who received lymphadenectomy than those who did not receive lymphadenectomy, with an average OS of 36 months and 12 months, respectively (*p* = 0.001; Figure 2D).To exclude the possible confounding effect caused by the uneven distribution of clinical features, the stage, age, and metastasis of lymph nodes of the patients were examined; the results demonstrated that there was no great variation in these factors (Table S4). Next, we compared the median OS of patients who had surgery (RH, TAH) with those who had adjuvant treatment and those who did not, and observed that among patients who had surgery, those who received other therapies had significantly better outcomes than those who did not, with an average OS of 36.5 months and 12 months, respectively (*p* = 0.051; Figure 3A).

**FIGURE 4:**
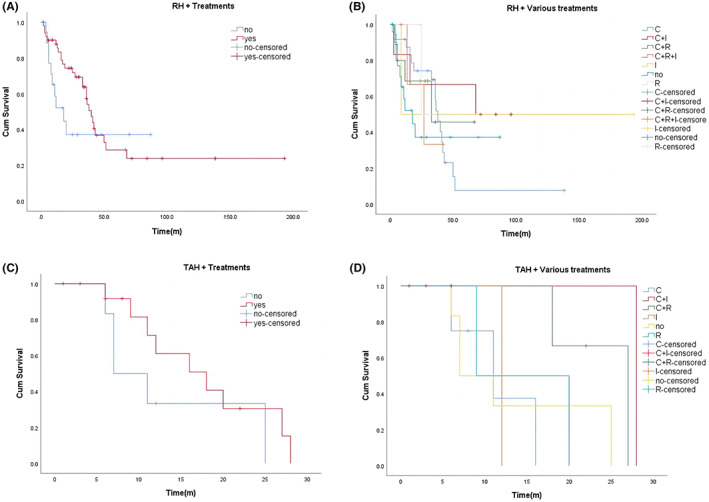
(A) Kaplan–Meier survival curves for OS in patients of RH‐based surgery with and without other treatments. Among patients undergoing RH‐based surgery, the median OS was significantly lower in patients without adjuvant therapy than in patients with adjuvant therapy. (B) Kaplan–Meier survival curves for OS in patients of RH‐based surgery plus various treatments vs. no other treatments. There was no significant difference in median OS between patients who underwent RH‐based surgery with the addition of different adjuvant treatments (*p* = 0.819). (C) Kaplan–Meier survival curves for OS in patients of TAH‐based surgery with and without other treatments. Patients who received adjuvant therapy had a higher median OS than those who had TAH‐based surgery alone, 18 months versus 7 months, respectively. (D) Kaplan–Meier survival curves for OS in patients of TAH‐based surgery plus various treatments vs. no other treatments. The median OS of chemotherapy plus immunotherapy and chemotherapy plus radiotherapy was 28 months and 27 months, while the median OS of chemotherapy, radiotherapy and immunotherapy alone was 11 months, 9 months, and 12 months.

We also considered the possible influence of confounding factors (stage, age, and lymph node metastasis), and the findings also indicated a non‐significant variation between their clinical characteristics (Table S5). There was no significant variation in median OS among patients receiving different adjuvant treatments (*p* = 0.548; Figure 3B); however, it can be seen in the figure that the average survival time of chemotherapy plus immunotherapy (median OS: 68 months) was the longest, followed by chemotherapy and chemotherapy plus radiotherapy. In addition, among patients that underwent RH‐based surgery, we observed that those who did not receive adjuvant therapy had a significantly lower average OS than those who received adjuvant therapy, with an average OS of 18 months and 40 months, respectively, but this was not statistically significant (*p* = 0.240; Figure 4A). We detected no significant variation in OS among patients who received different adjuvant treatments based on RH (*p* = 0.819; Figure 4B), and the result was similar to that in Figure 3B. Among patients with TAH‐based surgery, we found a variation in median survival among patients who had adjuvant therapy and those who did not with those who had adjuvant therapy having a higher average survival than those who received TAH‐based surgery alone (*p* = 0.222; Figure 4C). Moreover, we observed a non‐significant variation in survival among patients receiving different adjuvant treatment regimens (*p* = 0.188; Figure 4D); these results were similar to those in Figures 3B and 4B. In univariate and multivariate Cox regression models, only FIGO Stage IV was consistent and could serve as a separate risk factor (Table 2). In Table 2, analysis outcomes showed that age, and FIGO Stage II, III, and IV, increased the risk of death. At the same time, RH, TAH, lymphadenectomy and adjuvant therapy were correlated with a decreased risk of mortality.

**TABLE 2:** Univariate and multivariate cox proportional hazards regression analysis.

Characteristics	Univariate analyzes		Multivariate analysis	
	HR (95% CI)	*p* value	HR (95% CI)	*p* value
Age	1.011 (0.992–1.030)	0.245	1.346 (0.819–2.213)	0.241
Stage I	1		1	
II	1.563 (0.817–2.989)	0.177	1.967 (0.930–4.162)	0.077
III	3.022 (1.443–6.331)	0.003	2.038 (0.832–4.995)	0.120
IV	7.112 (2.918–23.019)	0.001	4.070 (1.118–14.808)	0.033
No surgery	1		1	
RH	0.271 (0.160–0.460)	0.002	0.427 (0.174–1.051)	0.064
TAH	0.661 (0.334–1.311)	0.236	0.824 (0.372–1.814)	0.633
No lymph node metastasis	1		1	
Lymph node metastasis	0.588 (0.222–1.557)	0.286	5.282 (0.873–31.972)	0.070
No lymphadenectomy	1		1	
lymphadenectomy	0.438 (0.276–0.693)	0.001	0.249 (0.033–1.905)	0.180
No treatments or only surgery	1		1	
Treatments other than surgery	0.638 (0.383–1.063)	0.084	0.519 (0.291–0.926)	0.027

## 4 DISCUSSION

PMMC is a very rare disease and, to date, specific risk factors leading to susceptibility have not been identified. Therefore, detection and diagnosis of this disease are still difficult. Cervical melanoma originates from cervical melanocytes,^79^ but these may be non‐pigmented, and if so, it is easy to be misdiagnosed. Moreover, there is a need to differentiate it from other diseases such as rhabdomyosarcoma, leiomyosarcoma, adenocarcinoma, squamous cell carcinoma, and malignant peripheral schwannoma.^80^, ^81^, ^82^ The diagnosis of PMMC is usually based on gynecological examination, colposcopy, histopathological examination, and immunohistochemical staining. In 2003, the American Joint Committee on Cancer (AJCC) revised TNM staging of melanoma; it now incorporates multiple prognostic factors such as thickness of primary (T) melanoma, existence of ulceration, lymph node metastasis (N), and distant metastasis (M).^83^ However, because the pattern and clinical symptoms of PMMC are similar to those of cervical cancer, and the FIGO staging system for cervical cancer has a good correlation with its prognosis, most authors accept the use of FIGO staging as a basis for the staging of PMMC.^84^, ^85^ Pusceddu^86^ showed that the 5‐year survival rate of primary cervical melanoma was 18.8% in FIGO Stage I, 11.1% in Stage II, and 0 in Stages III and IV, indicating that the FIGO stage is clearly related to prognosis. This is in line with the results of our research; with the increase of the FIGO stage, the median survival time also gradually decreased, suggesting that the use of FIGO staging for PMMC is more meaningful than TNM staging.

There are currently no clear guidelines for the treatment of PMMC. We checked the guidelines for cervical cancer and none of them specifically mentioned the diagnosis and treatment of PMMC. Although there is no agreement on the appropriate treatment for PMMC, surgery is still the default option.^87^ The surgical approach is often a radical hysterectomy, with or without bilateral salpingectomy and oophorectomy, lymph node dissection, and partial vaginal resection. In the current study, the vast majority of patients underwent RH‐based surgery and they had a longer median survival time than patients without surgery or TAH‐based surgery; therefore, RH‐based surgery is recommended for PMMC. Radiation therapy is mainly used for those patients who are unsuitable for surgery, for palliative treatment of advanced patients, or as adjuvant therapy and neoadjuvant therapy.^75^ It can also be used as preoperative radiotherapy in locally advanced patients to reduce tumor size, so that conservative surgery can then be implemented.^88^ In our study, radiotherapy was mostly used in combination with chemotherapy. Chemotherapy agents such as dacarbazine have been used in cases of advanced gynecologic melanoma disease to reduce tumor size with an effective rate of approximately 15%–20%.^89^, ^90^ Based on this principle, dacarbazine is the most common chemotherapy medication for PMMC, and it is often combined with cisplatin, vincristine, or bleomycin.

In the current study, most patients were treated with chemotherapy. In the treatment of melanoma, immune‐targeted therapy continues to make progress, with the most prominent being immune checkpoint inhibitors. Currently, most research is aimed at two types of targets: cytotoxic T lymphocyte antigen‐4 (CTLA‐4) and programmed death receptor 1 (PD‐1) pathways. Several articles report the effectiveness of the anti‐PD‐1 antibody drug nivolumab in primary cervical and vaginal MM.^91^, ^92^, ^93^ In this study, we discovered a non‐significant variation between various adjuvant regimens, including chemotherapy, radiotherapy, and immunotherapy, possibly because the sample size of some regimens was too small. However, compared with patients who did not receive adjuvant therapy, the survival time with adjuvant therapy was significantly longer. However, Min et al.^94^ revealed that the survival time of patients with RH‐based surgery supplemented with adjuvant therapy was shorter than that without adjuvant therapy, which was contrary to the conclusion reached in the current study, the latter being different from expectations. The reason behind potential bias in the Min et al. study may have been that the number of lymph node metastasis patients was significantly different between the two groups (with lymph node metastasis and without lymph node metastasis), and the median survival time of lymph node metastasis patients was significantly lower than that of those without lymph node metastasis. In addition, the sample size of patients who added adjuvant therapy was not sufficiently large, which may also have led to bias in the results. The current study concluded that lymph node metastasis patients had a better prognosis than those without lymph node metastasis. As shown in Table S1, among the 165 cases, there were 35 with no lymph node metastasis and 18 cases with lymph node metastasis information. Nearly 50% of the patients were still alive at the time of the survey, so an effective survival period could not be obtained. Several cases with lymph node metastases were seen to have longer overall survival; this may be because the valid sample size was too small, and gave unexpected results. Exploring the effects of adjuvant therapy other than surgery on patients' prognosis has been limited. It is therefore necessary to design studies with larger sample sizes to explore other complementary treatments besides surgery. Further biological and clinical studies are needed to determine a more appropriate and effective standard of care. In addition, the staging system regarding survival and prognosis remains a controversial issue. It is expected that relevant scientific societies will create a new staging system for PMMC to better correlate disease stage and patient prognosis. It is hoped that controversies regarding staging, diagnosis, and treatment procedures will be resolved in the near future.

In summary, this is the first comprehensive analysis of 165 cases of PMMC in the Chinese population, which has a high incidence of cervical cancer and a large sample size compared with other countries.^95^ This study showed that the survival time of patients with Stages III and IV was shorter than those of Stages I and II, and surgery combined with adjuvant therapy was helpful in improving survival time. Early diagnosis and treatment are extremely important for the prognosis and survival of patients. Because no standardized protocols or treatment guidelines have been published for this rare cancer, clinicians still refer to the standard of cervical squamous cell carcinoma for surgery in PMMC patients. According to this review of past cases, the current common and effective treatment plan is radical surgical resection combined with lymph node resection and other adjuvant therapy according to individual circumstances. This has guiding significance for clinical treatment, and also confirms from the side that the current treatment of PMMC still refer to the cervical squamous cell carcinoma, but the preoperative and postoperative adjuvant therapy still needs to be further explored.

## AUTHOR CONTRIBUTIONS

**Zhen Ma:** Methodology (equal). **Yumei Chen:** Methodology (equal). **Shibo Zhao:** Methodology (equal). **Caiyun Chen:** Methodology (equal).

## FUNDING INFORMATION

This study was surpported by Study on the regulatory effect and mechanism of AGRN in malignant biological behavior of cervical cancer (2022A703‐3).

## CONFLICT OF INTEREST STATEMENT

All authors declare no financial interest and no conflicts of interest.

## ETHICS STATEMENT

This study involving human volunteers and did not require an ethical assessment and approval in compliance with the local legislation and institutional requirements. In compliance with national legislation and institutional regulations, this study did not need written informed consent for participation.

## Supporting information


**Table S1.** **Table S2 Table S3 Table S4 Table S5**

## Data Availability

The datasets generated during and/or analyzed during the current study are available from the corresponding author on reasonable request.
