# Supplementary material for: Primary malignant melanoma of the cervix: A comprehensive analysis of case reports in the Chinese population
Source: Cancer Med. 2023 May 10;12(13):14052–61. doi: 10.1002/cam4.6054 (PMC10358241; doi:10.1002/cam4.6054)
Supplement: Supplementary file 1 — Table S1. Table S2 Table S3 Table S4 Table S5 [file CAM4-12-14052-s001.docx]

Supplementary Table 1 Main clinical features of 165 cases of PMMC

| Authors | Age | Symptom | Figo | Surgery | Lymph |
| --- | --- | --- | --- | --- | --- |
| 1966[8] | 56 | VB | NA | no | yes |
| 1986[9] | 41 | VB,VD | NA | RH+PLND+PV | yes |
|  | 47 | VB | NA | RH+PLND+PV | no |
| 1986[10] | 24 | VB | NA | TAH+Left adnexectomy | NA |
| 1987[11] | 41 | VB | NA | TAH | NA |
| 1989[12] | NA | NA | ⅡB | no | NA |
|  | NA | NA | NA | TAH+BSO | NA |
| 1990[13] | 72 | VD | NA | no | NA |
| 1991[14] | 35 | VB | NA | RH+PLND | no |
|  | 58 | VB | NA | RH+BSO+PLND | no |
| 1991[15] | 66 | VB | NA | NA | NA |
|  | 68 | VB | NA | NA | NA |
| 1991[16] | 30 | VB | ⅠB | RH+BSO+PV+LND | yes |
| 1991[17] | 62 | VB | Ⅲ | no | NA |
|  | 60 | VB | Ⅲ | RH | NA |
|  | 37 | VB | ⅠB | RH | NA |
| 1992[18] | 59 | VB,VD | Ⅱ | no | NA |
| 1992[19] | 49 | VD，VB | Ⅱ | yes not defined | NA |
| 1993[20] | 53 | VB | ⅡB | TAH+BSO | NA |
|  | 57 | VB | ⅡB | no | NA |
|  | 72 | VB | ⅡB | no | NA |
| 1994[21] | 60 | VB | NA | NA | NA |
|  | 70 | VB | NA | NA | NA |
|  | 24 | VB | NA | NA | NA |
| 1995[22] | 45 | VB | NA | NA | NA |
| 1996[23] | 25 | VB | NA | RH+BSO+PLND | yes |
| 1996[24] | 67 | VB | NA | no | NA |
| 1998[25] | 25 | VB | NA | RH+BSO+PLND | yes |
| 1999[26] | NA | NA | NA | RH+PLND | NA |
|  | NA | NA | NA | RH+PLND | NA |
| 2000[27] | NA | NA | NA | RH+BSO+LND | NA |
| 2000[28] | NA | VB | ⅡB | RH+BSO+PLND | no |
| 2000[29] | 61* | VD，VB | Ⅲ/Ⅳ/Ⅳ | no | NA |
| 2001[30] | 64 | VB | NA | NA | NA |
| 2001[31] | 53 | VB | NA | TAH+BSO | NA |
|  | 60 | VB | NA | TAH+BSO+PLND+TV+Partial urethral resection | NA |
|  | 51 | VD | NA | no | NA |
| 2002[32] | 55 | VB | ⅡB | RH+PLND | yes |
| 2002[33] | 55/59 | Hematuria/VB | NA | TAH+LND | no |
| 2003[34] | 40 | VB | NA | RH+PLND | yes |
| 2004[35] | 67 | VB | NA | no | NA |
| Supplementary Table 1 Main clinical features of 165 cases of PMMC(continued) | | | | | |
| 2004[36] | NA | VB | Ⅰ/Ⅱ/Ⅱ/Ⅱ | RH+PLND+PV | NA |
| 2005[37] | 55 | no | Ⅰ | RH+PLND | no |
|  | 47 | VD | Ⅲ | no | NA |
| 2005[38] | 33 | VB | ⅢB | RH+PLND | yes |
|  | 53 | VB | ⅠB | RH+PLND | NA |
|  | 40 | VD | ⅠB | RH+PLND | NA |
|  | 54 | VD | ⅠB | RH+PLND+TV | NA |
|  | 37 | VD | NA | RH+PLND+TV | yes |
| 2005[39] | 45* | Postcoital VB and VD | NA | RH+PLND | NA |
| 2005[40] | 40 | VB | NA | TAH+BSO+PLND | no |
| 2007[41] | 38 | VB | ⅠB1 | RH+BSO+PLND | no |
| 2007[42] | 44 | VD | Ⅰ | RH+BSO+PLND | no |
| 2007[43] | NA | NA | Ⅰ/Ⅱ/Ⅱ/Ⅱ/Ⅱ | no/RH/RH/RH/RH | NA |
| 2008[44] | 36 | VB | NA | TAH+Right adnexal excision | NA |
| 2008[45] | 64 | VB | NA | TAH+TV | no |
| 2008[46] | 28 | VB | ⅡA | RH+BSO+PLND+Total vaginal vulvotomy | NA |
|  | 67 | VB | ⅡA | TAH+BSO+PLND+PV | NA |
| 2008[47] | 58 | VB | ⅠB | RH+PLND | yes |
|  | 51 | VB,VD | Ⅳ | no | no |
| 2008[48] | 40 | VB | NA | NA | no |
| 2009[49] | 41 | VB | ⅠB2 | RH+PLND | no |
| 2009[50] | 60 | VB | NA | NA | NA |
| 2009[51] | 50 | VB | NA | TAH+BSO | NA |
| 2009[52] | 58 | VB | ⅡA | RH+PLND | NA |
|  | 63 | VB | ⅡA | RH+PLND | NA |
|  | 60 | VB | ⅢA | RH+PLND+TV | NA |
|  | 51 | VD | Ⅳ | no | NA |
|  | 53 | VB | ⅢA | no | NA |
|  | 61 | VD | ⅡA | RH+PLND | NA |
|  | 55 | Cervical vegetations | ⅠB1 | RH+PLND | NA |
|  | 47 | VD | ⅢB | no | NA |
| 2009[53] | 72 | VB | NA | TAH+BSO | NA |
| 2009[54] | 67 | VB | ⅡA | TAH+BSO+PLND+PV | no |
| 2010[55] | 46 | VB | NA | RH+PLND | no |
| 2010[56] | 75 | VB,VD | Ⅱ | yes | no |
|  | 56 | VB,VD | Ⅱ | no | no |
|  | 51 | VB,VD | Ⅰ | yes not defined | no |
|  | 70 | VB,VD | Ⅰ | yes not defined | no |
| 2011[57] | 59 | VB | NA | RH+PLND | NA |
| 2011[58] | 67 | VB | ⅠB1 | RH+BSO+PLND+PV | no |
| Supplementary Table 1 Main clinical features of 165 cases of PMMC(continued) | | | | | |
| 2012[59] | 51 | VB | NA | no | NA |
|  | 55 | Contact VB | NA | RH+PLND | NA |
|  | 28 | Contact VB | NA | RH+PLND | NA |
|  | 83 | VB | NA | RH+PLND | NA |
|  | 60 | VB | NA | RH+PLND | NA |
|  | 43 | VD | NA | RH+PLND | NA |
| 2012[60] | 59 | VB | NA | RH+PLND | no |
| 2012[61] | 59 | VB | ⅠA | no | NA |
| 2012[62] | 19 | NA | Ⅱ | no | NA |
| 2012[63] | NA | NA | ⅡB1 | RH+BSO+PLND | NA |
| 2013[64] | 35 | Cervical nodes | NA | TAH+BSO+PLND+PV | yes |
| 2013[65] | 49 | VB,VD | ⅡA | RH+BSO+PLND | no |
| 2014[66] | 46 | VB | NA | TAH+BSO+PLND | no |
|  | 58 | VB | NA | TAH+BSO+PLND | NA |
| 2014[67] | 67 | VD | ⅡA | RH+BSO+PLND+Para-aortic lymph node sampling | no |
| 2014[68] | 65 | VB | ⅠB1 | RH+BSO+PLND | no |
| 2014[69] | 64 | VD | NA | NA | NA |
| 2015[70] | 51 | VB | ⅠB1 | RH+BSO+PLND | no |
|  | 64 | VB | ⅡA | RH+BSO+PLND+TV | no |
|  | 56 | Contact VB | ⅢA | RH+BSO+PLND | no |
|  | 72 | VB | ⅢA | TAH+TV+BSO+Vaginal atresia | no |
| 2017[71] | 53 | VB | NA | no | NA |
| 2017[72] | 69 | VB,VD | NA | RH+BSO+PLND | no |
|  | 42 | NA | NA | RH+BSO+PLND | yes |
|  | 47 | NA | NA | RH+BSO+PLND | no |
|  | 64 | VB,VD | NA | RH+BSO+PLND | yes |
|  | 67 | VB,VD | NA | no | yes |
|  | 73 | VB,VD | NA | no | no |
| 2017[73] | 61 | VB | ⅡB | RH+PLND | NA |
|  | 74 | VB | ⅠB2 | RH+PLND | NA |
|  | 56 | VB | ⅢB | No | NA |
|  | 74 | VB | ⅡA1 | TAH+PLND | NA |
|  | 77 | VB | ⅠB1 | TAH | NA |
|  | 45 | VB | ⅠB1 | RH+PLND | NA |
|  | 50 | VB | ⅠB2 | TAH+PLND | NA |
|  | 58 | VB | ⅠB1 | RH+PLND | NA |
|  | 57 | VB | ⅡA1 | No | NA |
|  | 42 | VB | ⅡB | TAH | NA |
|  | 63 | VB | ⅢB | No | NA |
|  | 54 | VB | ⅡB | RH+PLND | NA |
| Supplementary Table 1 Main clinical features of 165 cases of PMMC(continued) | | | | | |
|  | 78 | VB | ⅢB | No | NA |
|  | 68 | VB | ⅡA1 | RH+PLND | NA |
| 2018[74] | 56 | VB | ⅠB1 | RH+BSO | no |
|  | 62 | VB | ⅡB | RH+BSO | yes |
|  | 38 | VB | ⅣB | no | yes |
|  | 62 | VB | ⅠB1 | RH+BSO | NA |
|  | 53 | VB | ⅠB1 | RH+BSO+Argon heliumknife | NA |
|  | 57 | VD | ⅡB | No | NA |
|  | 80 | VB | ⅢB | No | NA |
|  | 54 | VB | ⅠB1 | RH+BSO | NA |
|  | 50 | VB | ⅡA2 | TAH | yes |
|  | 58 | VB | ⅡA | TAH | NA |
|  | 45 | VB | ⅠB1 | RH+BSO+PLND | yes |
|  | 55 | VB | ⅡB | RH+BSO | NA |
|  | 60 | VB | ⅠB1 | RH+BSO | no |
|  | 69 | Urinary incontinence | ⅠB1 | RH+vulva+local urethal resection | NA |
| 2019[75] | 55 | VB | ⅡA | RH+BSO+PLND | NA |
|  | 81 | VB | NA | no | NA |
| 2020[76] | 57 | VD | ⅡA | RH+BSO+PLND | NA |
|  | 65 | VB | ⅠB1 | RH+BSO+PLND | NA |
|  | 50 | VD | ⅡA | RH+BSO+PLND | NA |
|  | 58 | VB | ⅡA | RH+BSO+PLND | NA |
|  | 50 | Contact VB | ⅠB1 | RH+BSO+PLND | NA |
|  | 56 | VD | ⅢC1 | RH+BSO+PLND | NA |
| 2020[77] | 62 | VB | Ⅳ | RH+BSO+PLND+Pelvic lesion resection | no |
| 2021[78] | 53 | VB | Ⅱ | RH | NA |
|  | 54 | Contact VB | Ⅰ | RH | NA |
|  | 51 | VB | Ⅳ | no | NA |
|  | 56 | VB | Ⅱ | RH | NA |
|  | 61 | VB | Ⅱ | no | NA |
|  | 38 | VB | Ⅱ | no | NA |

Supplementary Table 1 Main clinical features of 165 cases of PMMC(continued)

| Authors | Chemotherapy | Radiotherapy | Immunotherapy | Survival(m) | Status | HPV infection |
| --- | --- | --- | --- | --- | --- | --- |
| 1966[8] | no | no | no | 4 | 1 | NA |
| 1986[9] | no | post-R | no | 6 | 0 | NA |
|  | pre-C | pre-R+post-R | pre-I(BCG) | 9 | 0 | NA |
| 1986[10] | no | no | no | 7 | 1 | NA |
| 1987[11] | no | no | no | 11 | 1 | NA |
| 1989[12] | no | R | no | 20 | 0 | NA |
|  | post-C | post-R | no | 22 | 0 | NA |
| Supplementary Table 1 Main clinical features of 165 cases of PMMC(continued) | | | | | | |
| 1990[13] | no | no | no | 2 | 1 | NA |
| 1991[14] | no | no | no | 17 | 0 | NA |
|  | no | no | no | 5 | 0 | NA |
| 1991[15] | NA | NA | NA | NA | NA | NA |
|  | NA | NA | NA | NA | NA | NA |
| 1991[16] | no | post-R | no | 34 | 0 | NA |
| 1991[17] | post-C | no | no | 14 | 1 | NA |
|  | post-C | pre-R | no | 12 | 1 | NA |
|  | no | no | no | 10 | 0 | NA |
| 1992[18] | C | R | no | 12 | 1 | NA |
| 1992[19] | NA | NA | NA | NA | NA | NA |
| 1993[20] | pre-C+post-C | post-R | no | 27 | 1 | NA |
|  | no | R | no | NA | NA | NA |
|  | no | no | no | NA | NA | NA |
| 1994[21] | NA | NA | NA | NA | NA | NA |
|  | NA | NA | NA | NA | NA | NA |
|  | NA | NA | NA | NA | NA | NA |
| 1995[22] | NA | NA | NA | NA | NA | NA |
| 1996[23] | post-C | post-R | no | 30 | 0 | NA |
| 1996[24] | no | R | no | 9 | 1 | NA |
| 1998[25] | post-C | post-R | no | 36 | 0 | NA |
| 1999[26] | post-C | pre-R | no | NA | NA | NA |
|  | post-C | no | no | NA | NA | NA |
| 2000[27] | post-C | no | post-I(BCG) | NA | 3 | 1 |
| 2000[28] | post-C | post-R | no | 24 | 0 | NA |
| 2000[29] | NA | R/R/R | NA | 4/9/NA | 1/1/NA | NA |
| 2001[30] | NA | NA | NA | NA | NA | NA |
| 2001[31] | post-C | post-R | no | 18 | 1 | NA |
|  | post-C | no | no | NA | NA | NA |
|  | C | R | no | 3 | 1 | NA |
| 2002[32] | post-C | no | no | NA | NA | NA |
| 2002[33] | post-C | no | no | 6/11 | 1/1 | NA |
| 2003[34] | no | no | no | 48 | 0 | NA |
| 2004[35] | no | R | no | 7 | 1 | NA |
| 2004[36] | C/C/C/C | no/no/no/R | no/no/I/no | NA | NA | NA |
| 2005[37] | no | no | no | 12 | 1 | NA |
|  | C | no | no | 6 | 1 | NA |
| 2005[38] | pre-C | no | no | 36 | 1 | NA |
|  | post-C | no | no | 41 | 1 | NA |
|  | no | no | no | 6 | 1 | NA |
| Supplementary Table 1 Main clinical features of 165 cases of PMMC(continued) | | | | | | |
|  | pre-C+post-C | no | pre-I(INF)+post-I(INF) | 72 | 0 | NA |
|  | pre-C+post-C | no | no | 44 | 0 | NA |
| 2005[39] | pre-C/post-C/no/post-C | pre-R/no/no/no | no/no/no/post-I | 42/42/6/84 | 0/1/1/0 | NA |
| 2005[40] | NA | NA | NA | NA | NA | NA |
| 2007[41] | no | post-R | no | 24 | 0 | NA |
| 2007[42] | post-C | no | post-I(INF) | NA | 0 | NA |
| 2007[43] | C/NA | R/NA | no/NA | 12/16/13/27/73 | 1/1/0/0/0 | NA |
| 2008[44] | post-C | no | post-I(INF) | 3 | 0 | NA |
| 2008[45] | post-C | no | post-I | 1 | 0 | NA |
| 2008[46] | post-C | no | no | 13 | 1 | NA |
|  | no | no | post-I(INF+IL-2) | 12 | 0 | NA |
| 2008[47] | no | no | no | 8 | 1 | NA |
|  | C | no | no | 5 | 1 | NA |
| 2008[48] | NA | NA | NA | NA | NA | NA |
| 2009[49] | pre-C+post-C | no | no | 12 | 0 | NA |
| 2009[50] | NA | NA | NA | NA | NA | NA |
| 2009[51] | post-C | no | no | 8 | 0 | NA |
| 2009[52] | post-C | post-R | post-I(IFN) | 27 | 1 | NA |
|  | post-C | no | no | 17 | 1 | NA |
|  | post-C | no | no | 19 | 1 | NA |
|  | C | no | no | 6 | 1 | NA |
|  | C | R | I(IFN) | 96 | 0 | NA |
|  | post-C | no | post-I(IFN) | 68 | 1 | NA |
|  | no | no | no | 11 | 1 | NA |
|  | C | no | no | 6 | 1 | NA |
| 2009[53] | no | no | no | 12 | 0 | NA |
| 2009[54] | post-C | no | post-I(IL-2+ INF) | 6 | 0 | NA |
| 2010[55] | no | no | no | 2 | 0 | NA |
| 2010[56] | post-C | no | no | 16 | 0 | NA |
|  | no | no | no | 6 | 1 | NA |
|  | post-C | no | no | 40 | 1 | NA |
|  | post-C | no | no | 46 | 0 | NA |
| 2011[57] | no | no | no | 18 | 1 | NA |
| 2011[58] | no | no | no | NA | NA | NA |
| 2012[59] | no | no | no | NA | NA | NA |
|  | no | no | no | 9 | 1 | NA |
| Supplementary Table 1 Main clinical features of 165 cases of PMMC(continued) | | | | | | |
|  | post-C | no | post-I | 96 | 0 | NA |
|  | no | no | no | 25 | 0 | NA |
|  | post-C | post-R | no | 4 | 1 | NA |
|  | no | no | no | 2 | 0 | NA |
| 2012[60] | no | no | no | 5 | NA | NA |
| 2012[61] | no | no | no | 8 | 0 | NA |
| 2012[62] | C | R | no | NA | 1 | NA |
| 2012[63] | no | no | no | NA | NA | NA |
| 2013[64] | NA | NA | NA | NA | NA | NA |
| 2013[65] | pre-C | post-R | no | 6 | 0 | yes |
| 2014[66] | no | no | no | 7 | 1 | NA |
|  | post-C | post-R | no | NA | NA | NA |
| 2014[67] | pre-C+post-C | no | post-I(INF) | NA | NA | NA |
| 2014[68] | pre-C+post-C | no | no | 30 | 0 | NA |
| 2014[69] | NA | NA | NA | NA | NA | NA |
| 2015[70] | no | post-R | no | 25 | 1 | NA |
|  | post-C | no | no | 40 | 1 | NA |
|  | post-C | no | no | 34 | 0 | NA |
|  | no | no | no | 6 | 1 | NA |
| 2017[71] | NA | NA | NA | NA | NA | NA |
| 2017[72] | post-C | no | no | 38 | 1 | NA |
|  | post-C | no | no | 36 | 1 | NA |
|  | post-C | no | no | 43 | 1 | NA |
|  | post-C | no | no | 50 | 1 | NA |
|  | C | no | I | 63 | 0 | NA |
|  | C | no | I | NA | NA | NA |
| 2017[73] | no | no | post-I(INF+IL-2) | 193 | 0 | NA |
|  | post-C | no | no | 33 | 1 | NA |
|  | post-C | post-R | no | 5 | 1 | NA |
|  | post-C | no | post-I(INF+IL-2) | 28 | 1 | NA |
|  | no | no | no | 25 | 1 | NA |
|  | no | no | no | 87 | 0 | NA |
|  | post-C | no | no | 16 | 1 | NA |
|  | post-C | no | no | 35 | 0 | NA |
|  | post-C | no | no | 4 | 1 | NA |
|  | no | pre-R | no | 9 | 1 | NA |
|  | post-C | post-R | no | 10 | 1 | NA |
|  | pre-C | pre-R | no | 33 | 1 | NA |
|  | post-C | post-R | no | 12 | 1 | NA |
| Supplementary Table 1 Main clinical features of 165 cases of PMMC(continued) | | | | | | |
|  | no | no | no | 20 | 1 | NA |
| 2018[74] | post-C | no | no | 36.5 | 1 | NA |
|  | post-C | post-R | post-I | 13.7 | 1 | NA |
|  | no | no | no | NA | NA | NA |
|  | no | no | no | 70 | 0 | NA |
|  | post-C | no | no | 51.5 | 1 | NA |
|  | post-C | no | post-I | 6 | 1 | NA |
|  | no | no | no | 3 | 1 | NA |
|  | no | no | no | NA | NA | NA |
|  | no | no | no | NA | NA | NA |
|  | no | post-R | no | 20 | 1 | NA |
|  | post-C | no | no | 3 | 1 | NA |
|  | pre-C | post-R | no | 5 | 1 | NA |
|  | no | no | no | 5 | 1 | NA |
|  | post-C | no | post-I | 16 | 1 | NA |
| 2019[75] | pre-C+post-C | pre-R | no | 67 | 0 | NA |
|  | no | no | no | 21 | 0 | NA |
| 2020[76] | post-C | no | no | 22 | 0 | NA |
|  | post-C | no | no | 138 | 0 | NA |
|  | no | post-R | post-I | NA | NA | NA |
|  | no | no | no | 4 | 1 | NA |
|  | no | no | no | 29 | 0 | NA |
|  | post-C | no | no | 2 | 1 | NA |
| 2020[77] | no | no | post-I(PD-1) | 9 | 0 | NA |
| 2021[78] | post-C | no | no | 16 | 1 | no |
|  | post-C | post-R | no | 28 | 0 | 阴性 |
|  | no | no | no | 3 | 1 | NA |
|  | no | no | no | NA | NA | NA |
|  | no | no | no | 15 | 1 | NA |
|  | C | R | no | 4 | 1 | 阴性 |

BSO:Bilateral salpingoophorectomy;INF:interferon;IL-2: interleukin-2;TAH: Total hysterectomy; PLND: Pelvic lymphadenectomy; PV: Partial vaginectomy; pre-C:Preoperative chemotherapy; post-C: Postoperative chemotherapy; pre-R:Preoperative radiotherapy; post-R:Postoperative radiotherapy; post-I:Postoperative immunotherapy;RH: Radical hysterectomy;TV: Total vaginectomy ;VB:Vaginal bleeding; VD:Vaginal discharge;

Supplementary Table 2 Comparison of clinical characteristics of patients with different FIGO stages

|  | StageⅠ | StageⅡ | StageⅢ | StageⅣ | X^2^ | P |
| --- | --- | --- | --- | --- | --- | --- |
| Treatment(yes） | 21 | 34 | 13 | 4 |  |  |
| Treatment（no） | 13 | 8 | 2 | 2 | 5.146 | 0.161 |
| Age（>60) | 9 | 13 | 8 | 2 |  |  |
| Age（<60) | 23 | 24 | 7 | 4 | 2.856 | 0.414 |
| Lymphadenectomy(yes) | 19 | 23 | 4 | 1 |  |  |
| Lymphadenectomy(no) | 21 | 22 | 11 | 5 | 4.758 | 0.19 |
| Lymphmetastasis(yes) | 3 | 3 | 1 | 1 |  |  |
| Lymphmetastasis(no) | 11 | 7 | 2 | 2 | 0.39 | 0.942 |

Supplementary Table 3 Comparison of clinical characteristics of patients without surgery and those who received RH- or TAH-based surgery

|  | NO | RH | TAH | X^2^ | P |
| --- | --- | --- | --- | --- | --- |
| StageⅠ | 2 | 28 | 2 |  |  |
| StageⅡ | 10 | 28 | 7 |  |  |
| StageⅢ | 9 | 5 | 1 |  |  |
| StageⅣ | 5 | 1 | 0 | 28.618 | 0.001 |
| Age（>60) | 14 | 21 | 8 |  |  |
| Age（<60) | 20 | 55 | 16 | 1.998 | 0.368 |
| Lymphmetastasis(yes) | 3 | 13 | 2 |  |  |
| Lymphmetastasis(no) | 3 | 22 | 7 | 1.272 | 0.529 |

Supplementary Table 4 Comparison of clinical characteristics of patients with and without lymphadenectomy

|  | Lymphadenectomy(yes) | Lymphadenectomy(no) | X^2^ | P |
| --- | --- | --- | --- | --- |
| StageⅠ | 19 | 21 |  |  |
| StageⅡ | 23 | 22 |  |  |
| StageⅢ | 4 | 11 |  |  |
| StageⅣ | 1 | 5 | 4.758 | 0.19 |
| Lymphmetastasis(yes) | 13 | 5 |  |  |
| Lymphmetastasis(no) | 25 | 8 | 0.039 | 0.787 |
| Age（>60) | 20 | 23 |  |  |
| Age（<60) | 54 | 38 | 1.756 | 0.185 |

Supplementary Table 5 Comparison of clinical characteristics of patients with surgery alone and patients with surgery combined with adjuvant therapy

|  | Surgery | Surgery +Treatments | X^2^ | P |
| --- | --- | --- | --- | --- |
| StageⅠ | 12 | 20 |  |  |
| StageⅡ | 5 | 27 |  |  |
| StageⅢ | 1 | 5 |  |  |
| StageⅣ | 0 | 1 | 4.674 | 0.197 |
| Age（>60) | 8 | 23 |  |  |
| Age（<60) | 23 | 47 | 0.502 | 0.479 |
| Lymphmetastasis(yes) | 3 | 11 |  |  |
| Lymphmetastasis(no) | 9 | 22 | 0.285 | 0.593 |
